# Racing Experiences of Recreational Distance Runners following Omnivorous, Vegetarian, and Vegan Diets (Part B)—Results from the NURMI Study (Step 2)

**DOI:** 10.3390/nu15102243

**Published:** 2023-05-09

**Authors:** Katharina Wirnitzer, Derrick Tanous, Mohamad Motevalli, Karl-Heinz Wagner, Christian Raschner, Gerold Wirnitzer, Claus Leitzmann, Thomas Rosemann, Beat Knechtle

**Affiliations:** 1 Department of Research and Development in Teacher Education, University College of Teacher Education Tyrol (PH Tirol), 6010 Innsbruck, Austria; 2 Department of Sport Science, University of Innsbruck, 6020 Innsbruck, Austria; 3 Research Center Medical Humanities, University of Innsbruck, 6020 Innsbruck, Austria; 4 Department of Nutritional Sciences, University of Vienna, 1090 Vienna, Austria; 5 adventureV & change2V, 6135 Stans, Austria; 6 Institute of Nutrition, University of Gießen, 35390 Gießen, Germany; 7 Institute of Primary Care, University of Zurich, 8091 Zurich, Switzerland; 8 Medbase St. Gallen, Am Vadianplatz, 9001 St. Gallen, Switzerland

**Keywords:** diet type, plant-based, exercise training, endurance, running, marathon, half-marathon, competition

## Abstract

The potential running or endurance performance difference based on following different general types of diets, such as omnivorous, vegetarian, or vegan, remains questionable. Several underlying modifiable factors of long-distance running performance, especially runner training behaviors and experience, diminish the clarity of results when analyzing dietary subgroups. Based on the cross-sectional design (survey), the NURMI Study Step 2 aimed to investigate a plethora of training behaviors among recreational long-distance running athletes and the relationship of general diet types with best time race performance. The statistical analysis was based on Chi-squared and Wilcoxon tests. The final sample (*n* = 245) included fit recreational long-distance runners following an omnivorous diet (*n* = 109), a vegetarian diet (*n* = 45), or a vegan diet (*n* = 91). Significant differences were found between the dietary subgroups in body mass index (*p* = 0.001), sex (*p* = 0.004), marital status (*p* = 0.029), and running-related motivations for well-being (*p* < 0.05) but not in age (*p* = 0.054). No significant difference was found for best time half-marathon, marathon, and/or ultra-marathon race performance based on diet type (*p* > 0.05). Whether the vegan diet is associated with enhanced endurance performance remains unclear. Although, the present results are suggestive that 100% plant-based (vegan) nutrition is compatible with distance running performance at the least.

## 1. Introduction

The resulting beneficial effects of long-distance runner training behaviors on individual anatomy (body weight maintenance, body mass index, body composition, and muscle fiber assortment adaptations) [1,2], physiology (fat metabolism, blood ejection fraction, mitochondrial density, capillarization) [3,4], and mentality [5] have been studied previously. Specific types of training, such as low-intensity long bouts of running, high-intensity interval training, or tapering, have been shown to result in distinct health and performance benefits [3,4]. Due to safety (e.g., injury avoidance) and performance enhancement, the specific runner training frequencies, intensities, durations, and types are often adhered to by a periodization outline up until and after the main racing event [6,7,8]. Furthermore, through the recreational distance runner lifestyle, the accumulation of years of running and racing experience likely provides advanced benefits for health (heart efficiency, healthy body weight) [4] and performance (pacing strategy, mentality, focus) alike [6,9]. 

Health appears to be very closely related to physical performance [10], and diet type is known to correlate with many different factors of health, including but not limited to micronutrient status [11], lifestyle behaviors [12,13], risk of chronic disease [14], and all-cause mortality [15]. The proportion of a wide variety of plant-based whole foods (fruits; vegetables; flowers, roots, legumes, nuts; whole grains) making up an individual’s diet has been suggested to enhance running performance, particularly due to being a rich source of unrefined carbohydrates and antioxidants [16,17,18]. Western omnivorous and vegetarian diets appear to have similar compositions of plant-based whole foods [19], although a higher consumption of healthy foods among vegetarians is not uncommon [20]. Thus, meat consumption may be the only major dietary difference between omnivorous and vegetarian diets [21]. The lack of significant proportional differences in macronutrient consumption (carbohydrate, protein, fat) between omnivores and vegetarians—primarily due to the consumption of milk and dairy products—may explain why no running performance differences have been found between these diets in the previous research [22,23]. The vegan diet (100% plant-based nutrition), however, excludes the majority of food products typically consumed in a Western omnivorous diet [21], which may explain why significant health benefits have been associated with vegans [11,15,23,24,25,26]. Likewise, the macronutrient distribution from the vegan diet is exceptionally reliant on carbohydrates, with the possibility of safely consuming up to 95% of daily calories from carbohydrates during multiple-day, moderate-to-vigorous intensity endurance performance [27]. With the appropriate dietary planning, the largest nutritional organization around the world—the Academy of Nutrition and Dietetics—supports adherence to the vegan diet for health and sports performance [21].

The topic of differences between diet types in endurance performance has been considered and examined for over 100 years [28]. The issue on the vegan diet has been much less studied [17] than the vegetarian diet [29], and the omnivorous diet—the world’s most common diet type [30]—typically serves as the reference group for comparisons [31]. What remains unclear from the scientific evidence to date [18,27,32,33,34,35,36,37,38] is whether a running performance difference exists when including the vegan diet as an additional comparator group. Due to the vast influence of several different factors in running performance, such as genetics [39] or personal training behavior [40,41], observable diet type differences may be limited [36,37]. Previous population studies on long-distance recreational runners comparing omnivorous, vegetarian, and vegan diet types in performance have only examined a limited extent of training variables, such as weekly exercise frequency, running distance, durations, and/or professional support [32,33,35]. However, motivations for running and racing have been shown to impact training behaviors in recreational runners [42] and are therefore imperative to consider for best time racing performance.

Therefore, based on the inconclusive evidence on the comparison of endurance performance among recreational long-distance runners following omnivorous, vegetarian, and vegan diets [18,32,33,35,36,37], this study is the first to examine different general diet types among runners regarding long-distance athletic performance in recreational half-marathon (HM), marathon (M), and ultra-marathon (UM) races while considering a comprehensive list of training behaviors. The present investigation primarily aimed to assess performance while considering a thorough list of training and experience confounders secondarily. It was hypothesized that there is no difference in best HM and M time performances between recreational long-distance athletes based on adhering to general omnivorous, vegetarian, or vegan diets. 

## 2. Materials and Methods

The NURMI study (Nutrition and Running High Mileage) was a cross-sectional (survey-based) investigation on recreational distance runners (10 km, HM, M, and UM distances) and was planned out in three separate steps. The present article (Part B) is seamlessly sequenced to the complete methodological details [43,44,45,46,47,48] and results previously published in Part A [49] and thus includes only the limited methods regarding the present results. The study protocol was accepted (May 2015; EKSG 14/145) by the ethics board in St. Gallen, Switzerland [50], and a trial registration was completed (ISRCTN73074080). The NURMI study subjects were informed of the procedure in writing prior to enrollment and submitted their informed consent.

**Figure 1:**
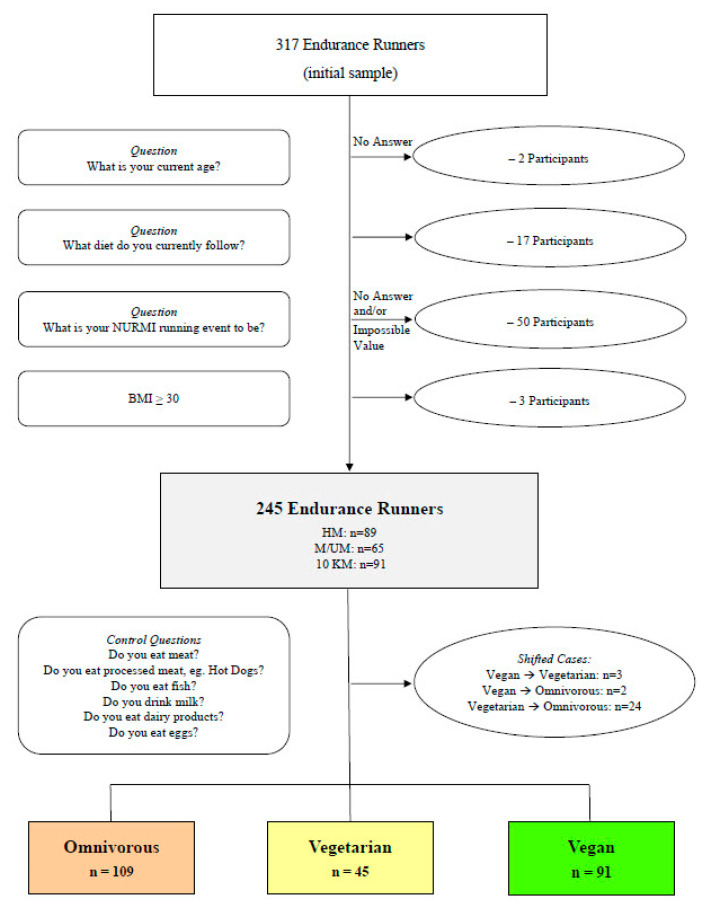
Flow of Subjects’ Dietary Subgroup Classification and Enrollment. BMI—body mass index. HM—half-marathon. M/UM—marathon/ultra-marathon. 10 km—10 kilometers.

Figure 1 displays the subjects’ dietary subgroup and flow of enrollment. Part A of the present investigation includes additional details of subject recruitment, inclusion criteria, and study procedures [49]. Further subject characteristics are displayed by Table 1.

The subjects’ training details and race performances were described based on the diet types related to prespecified running-related variables: total number of finished races, distance of first race (10 km, HM, M/UM), best race time (HM, M, UM), running history (complete years of active running without break and the years of age at the first race), training behaviors (number of weekly runs with weekly and daily distances covered (km) and durations (hours) for (i) Training Type C and (ii) Training Type D; weekly km of Training Type A; qualified professional support; and overall training duration (months)), and racing experiences (the number of HM and M races finished, the ratio of finished HM/M races to other races, and the number of finished races that were planned over the prior two years). Using an index for race performance with ten quantiles (range: 0–100), the best race times for HM and M distances were categorized separately (also for men and women) and transformed with a normalized aggregate mean and incorporated the number of races completed and age.

The statistical analysis was completed with R software (version 4.2.2 UCRT) [51]. Exploratory analysis was performed with descriptive statistics (median with interquartile rage (IQR) and means with standard deviation (SD)). Non-parametric testing was used to observe significant differences between dietary subgroups considering race experience (history) and running activity (training, etc.). Wilcoxon tests (ordinal/metric scales) and Chi-square tests (χ²; nominal scale) were used to verify the associations between variables. Multivariate linear regression was performed to examine the differences in well-being, hobby, and competition motivations, and regression analysis is shown as effect plots with a 95% confidence interval (95%-CI). The level of statistical significance was set at *p* ≤ 0.05.

## 3. Results

The survey was filled out and submitted by a total of 317 subjects, however, 72 did not fulfill the final inclusion criteria and were excluded from this investigation. Thus, 245 runners were included in the statistical analysis, with 104 males and 141 females mostly from Germany and Austria (*n* = 221; 90%), and some were from a variety of other countries (*n* = 24; 10%). Overall, the subjects were 39 years of age (IQR 17), had a body weight of 65 kg (IQR 14.2), a normal BMI (21.7 kg/m²; IQR 3.5), and were mostly married (*n* = 164; 67%). For diet type, there were 109 participants (36% of females; *n* = 51) following an omnivorous diet, 45 participants (18% of females; *n* = 26) following a vegetarian diet, and 91 participants (46% of females; *n* = 64) following a vegan diet.

Significant differences were found across the dietary subgroups for sex (*p* = 0.004), body weight (*p* = 0.001), and BMI (*p* = 0.001), where the omnivores were most likely to be male (*n* = 58; 53.2%) and the heaviest (68 kg; IQR 16.7) (*p* = 0.088). No significant differences were found for height (*p* = 0.088), country of residence (*p* = 0.171), race motive (*p* = 0.223), or preferred race distance (*p* = 0.483) based on diet type. A significant difference was found for academic qualifications across dietary subgroups (*p* = 0.029), the greatest proportion of omnivores had completed an upper secondary level of education (*n* = 41; 38%); most vegetarians had completed either upper secondary school (*n* = 17; 38%) or held a university degree or higher degree (*n* = 17; 38%), whereas the largest proportion of vegans had a university degree or higher (*n* = 33; 36% *p* = 0.029). The subjects’ characteristics with race motives, running preferences, and running and racing experience are provided in Table 1. Additional details are available elsewhere [49].

**Table 1:** Characteristics, Race Motives, and Running and Racing Experiences by Dietary Subgroups.

		Total	Omnivore	Vegetarian	Vegan	Statistics
		100% (245)	45% (109)	18% (45)	37% (91)	
**Age** **(years)**		39 (IQR 17)	43 (IQR 18)	39 (IQR 16)	37 (IQR 15)	F_(2, 242)_ = 2.95 *p* = 0.054
**BMI** **(kg/m²)**		21.7 (IQR 3.5)	22.6 (IQR 3.61)	20.8 (IQR 3.46)	21.3 (IQR 3.21)	F_(2, 242)_ = 7.03 *p* = 0.001
**Marital Status**	Divorced Married Single	6% (15) 67% (164) 27% (66)	3% (3) 75% (82) 22% (24)	4% (2) 58% (26) 38% (17)	11% (10) 62% (56) 27% (25)	χ^2^_(4)_ = 10.78 *p* = 0.029
**Race Distance**	10 km HM M/UM	37% (91) 36% (89) 27% (65)	34% (37) 36% (39) 30% (33)	33% (15) 44% (20) 22% (10)	43% (39) 33% (30) 24% (22)	χ^2^_(4)_ = 3.47 *p* = 0.483
**Race Motive**	Hobby Competition	46% (106) 54% (125)	50% (52) 50% (52)	34% (14) 66% (27)	47% (40) 53% (46)	χ^2^_(2)_ = 3.00 *p* = 0.223
**Favored Race Season**	Winter Spring Summer Fall	<1% (2) 46% (106) 23% (52) 31% (71)	<1% (1) 44% (46) 22% (23) 33% (34)	/ 44% (18) 27% (11) 29% (12)	1% (1) 49% (42) 21% (18) 29% (25)	χ^2^_(6)_ = 1.39 *p* = 0.966
**Complete Years of Active Running**		7 (IQR 7)	8 (IQR 10)	7 (IQR 6)	5 (IQR 8)	F_(2, 241)_ = 6.59 *p* = 0.002
**Years of Age at First Race**	10 km HM M UM Total	30 (IQ 16) 32 (IQR 16) 35 (IQR 13) 40 (IQR 11) 30 (IQR 16)	32 (IQ 18) 34 (IQR 18) 35 (IQR 12) 40 (IQR 10) 33 (IQR 19)	30 (IQ 15) 32 (IQR 15) 34 (IQR 17) 44 (IQR 10) 30 (IQR 16)	28 (IQ 15) 31 (IQR 14) 33 (IQR 12) 38 (IQR 10) 29 (IQR 14)	F_(2, 151)_ = 0.16 *p* = 0.853 F_(2, 216)_ = 0.89 *p* = 0.410 F_(2, 135)_ = 0.99 *p* = 0.374 F_(2, 45)_ = 1.31 *p* = 0.279 F_(2, 239)_ = 0.56 *p* = 0.527
**Distance of First Race**	10 km HM M	65% (157) 27% (65) 9% (21)	67% (72) 23% (25) 9% (10)	60% (27) 36% (16) 4% (2)	64% (58) 26% (24) 10% (9)	χ^2^_(4)_ = 3.19 *p* = 0.527
**Total Races Finished**		8 (IQR 11)	8 (IQR 14)	10 (IQR 12)	7 (IQR 8)	F_(2, 242)_ = 2.24 *p* = 0.109
**Ratio of Finished HM/M to Other Races**		40 (IQR 50)	40 (IQR 47)	33 (IQR 37)	47 (IQR 60)	F_(2, 242)_ = 0.35 *p* = 0.707
**Finished Planned Races (prior 2 years)**	HM M UM	2 (IQR 3) 1 (IQR 2) 0 (IQR 0)	2 (IQR 3) 1 (IQR 2) 0 (IQR 0)	2 (IQR 4) 0 (IQR 1) 0 (IQR 0)	2 (IQR 3) 0 (IQR 2) 0 (IQR 0)	F_(2, 242)_ = 1.17 *p* = 0.312 F_(2, 242)_ = 1.31 *p* = 0.273 F_(2, 242)_ = 1.21 *p* = 0.299
**Best Race Time (minutes)**	HM M UM	111 ± 33 230 ± 45 628 ± 489	106 ± 21 228 ± 45 705 ± 498	111 ± 25 231 ± 38 829 ± 806	118 ± 46 233 ± 48 454 ± 270	F_(2, 214)_ = 1.90 *p* = 0.153 F_(2, 129)_ = 0.22 *p* = 0.799 F_(2, 43)_ = 2.66 *p* = 0.081

Note. Data presented in median (IQR), percentage (%), total numbers, and mean ± SD. F statistic calculated by Wilcoxon test and χ2 statistic calculated by Pearson’s Chi-squared test. 10 km—10 kilometers. HM—half-marathon. M/UM—marathon/ultra-marathon.

### 3.1. Running History of Recreational Athletes

Spring was the favored race season of running competitions (*n* = 106; 46%), which was similar across dietary subgroups (*p* = 0.966). Similar racing experiences were found based on the dietary subgroups for (i) subject age at the first race, regardless of the distance (10 km, HM, M, or UM: *p* > 0.05); (ii) the distance (10 km, HM, M) of the first race (*p* = 0.527); (iii) the total number of races finished (*p* = 0.109); (iv) the ratio of finished HM/M races to other races (*p* = 0.707); (v) the finished planned races over the prior two years, regardless of the distance (10 km, HM, M, or UM: *p* > 0.05); and (vi) the best race time regardless of the distance (HM, M, or UM: *p* > 0.05). For running experience, a significant difference was found for the number of complete years of active running (*p* = 0.002), where omnivores had completed the most years (8 IQR 10) and vegans the least (5 IQR 8). 

**Figure 2:**
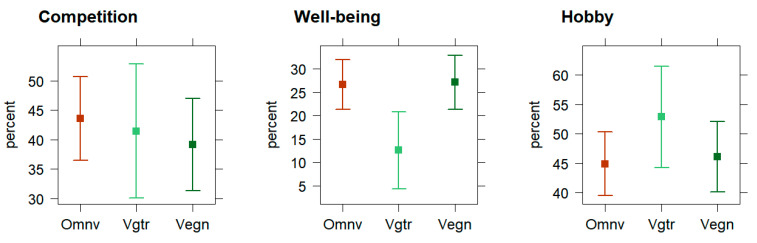
Effect plots display 95% CI average for training/running/racing motivations across dietary subgroups (*n* = 231): competition, well-being, and hobby. Note. 95% CIs were computed using the multivariate regression analyses (Wald approximation).

Multivariate linear regression showed a motivational difference across the dietary subgroups, where the omnivores were significantly more well-being motivated for running than the vegetarians (b = −12.6; 95% CI [−22.8–−2.47]; *p* < 0.05), as seen in Figure 2. The vegans were similarly well-being motivated for running compared to the omnivores (b = 1.88; 95% CI [−6.16–9.91]; *p* > 0.05). No dietary subgroup differences for competition (*p* > 0.05) or hobby motivations (*p* > 0.05) were observable in the multivariate linear regression.

### 3.2. Racing Experiences, Confounders, and Performance of Recreational Athletes

In predicting the best race time over HM and M distances, the linear regression analyses included the following confounders (displayed in Table 2): (1) the subjects’ running history (adjusted R^2^ = 0.16: complete years of active running and the years of age at the first race), with no significant differences found between omnivore and vegetarian (b = −1.66; 95% CI [−11.6–8.27]; *p* > 0.05) or vegan diets (b = 0.491; 95% CI [−7.76–8.74]; *p* > 0.05); (2) the subjects’ training behaviors (adjusted R^2^ = 0.22: Training Type C, Training Type D, weekly km of Training Type A, qualified professional support, and overall training duration), with no significant differences found between omnivore and vegetarian (b = −0.495; 95% CI [−10.5–9.51]; *p* > 0.05) or vegan diets (b = 1.85; 95% CI [−6.33–10]; *p* > 0.05); (3) the subjects’ racing experiences (adjusted R^2^ = 0.14: the number of HM and M races finished, the ratio of finished HM/M races to other races, and the number of finished races that were planned over the prior two years), with no significant differences found between omnivore and vegetarian (b = −0.716; 95% CI [−10.7–9.22]; *p* > 0.05) or vegan diets (b = 1.4; 95% CI [−6.78–9.59]; *p* > 0.05). 

## 4. Discussion

The present investigation, seamlessly sequenced to Part A [49], aimed to analyze performance differences of healthy and fit omnivorous, vegetarian, and vegan recreationally competitive runners while considering various training and experience confounders. The major findings were that in competitive distance runners (i) males were the most likely to follow an omnivorous diet and females were the most likely to follow a vegan diet; (ii) no significant differences were found between dietary subgroups in age, height, country of residence, training focus, race motive, preferred race distance, or preferred race season (iii) BMI was significantly different concerning diet type, with active and recreationally competitive omnivores having the highest BMI; (iv) no significant differences in racing history or best time performance of HM or M races between dietary subgroups were found; (v) no significant differences were found for best time performance of HM and M races based on the dietary subgroups while considering training and racing experience confounders.

Results from Part A of the present investigation indicated that no fundamental training difference exists between recreational distance runners capable of completing a half-marathon (at least) and following omnivore, vegetarian, or vegan diets [49]. Previously published results from the NURMI Study Step 1 (*n* = 2864), which was based on a general sample, have shown that diet type is a critical factor to consider regarding the best race performance of distance runners, attributed to 11–14% of the variation in the best HM and M time differences [35,36,37]. It is well-known that an individual’s diet or nutrition is vital for growth and to sustain life [52,53], which is essential in the advancement of human movement regarding superior sport performance [54]. However, the awareness of the vegan diet type in the role of physical performance is less common in the world of sports, although it is increasing [24]. 

This investigation was the first to consider a plethora of critical training confounders in best time race performance differences of recreational runners following different general diet types. When interpreting the best time race performance results, it is exceptionally noteworthy that several but not all impactful variables were similar across the dietary subgroups. Overall, the present sample exhibits comparably homogenous characteristics most importantly including age, height, participation in recreational sport, preferred race distance, and all specific training types [49]. However, important critical differences to consider for the best time race performance results were found concerning diet types, such as sex and BMI [55,56]. In connection, it has also been reported previously that additional variables not included in the present study are critical to consider regarding best race performance times such as genetics and specific anthropometrics, body composition and fat distribution, personal race day strategies (performance-enhancing substance usage, supplements, clothing/technology, etc.), environmental conditions (surfaces, profiles, such as elevation gain or loss, and degree of incline or decline), weather (severe or moderate, relative temperature and humidity, wind speed and direction), or time of day [3,35,37,47,48,57,58,59]. 

The present sample included a significant difference in sex based on following omnivorous, vegetarian, or vegan diets. A greater proportion of women than men reported to follow a vegan diet, which is consistent with previous findings [60]. Thus, sex differences are fundamental to consider when analyzing dietary subgroups. This investigation also found that a considerable proportion of highly motivated, physically active males followed vegetarian or vegan diets (46%) as compared to the general German population (3.07%) [61], which is also more apparent than in other countries [36]. The underlying motivations of male and female recreational runners to follow their specific self-reported diet type have been reported previously [43,62,63] and are primarily for ethical or health reasons [60]. 

The present investigation identified a significant difference in terms of academic qualification and diet type. As with previous reports [60], there is a link between higher education and vegetarian or vegan diet types especially. Likewise, this finding is most probably related to the greater proportion of females following vegan diets, who are consistently found to have extensive educational backgrounds [60]. In addition, it was found that the vegan participants were the most likely to be divorced, the vegetarians were most likely to be single, and the omnivorous participants were most likely to be married. However, the largest proportions within each diet type regarding marital status were married and living with their partner. The issue of the vegans being the most likely to be divorced may be primarily due to the divorce itself as a major life changing event, which has been reported to precede drastic dietary changes [64]. Likewise, the occurrence of a large proportion of single vegetarians may be related to the specific dietary lifestyle practices and the resultant cognitive dissonance [65]. The omnivorous participants, on the other hand, may neglect day-to-day lifestyle-related cognitive dissonance [65], which may prevent marital/relationship disturbances. 

The present sample shares remarkably similar training foci and race motives across dietary subgroups that likely set the foundation for the runners’ training behavior [42,47]. Dietary motivations [43], as well as the subjects’ initial running motivations and current running motivations, appear to be less important for influencing general or specific recreational runner training behaviors. Interestingly, subjects of the omnivorous and vegan dietary subgroups had a similar level of health interest regarding the current motivations for running, with vegetarians being the least interested. Therefore, recreational long-distance runners following omnivorous diets are referred to the scientific evidence on dietary-related cardiovascular risks of omnivorous nutrition [17,24,66,67]. Moreover, based on the dietary subgroups, the samples share a similar age of approximately 39 years and have been competing in various distance races (10 km, HM, M, UM) with similar increases in age for each successive and accentuating distance. Previous research has shown the importance of age in running performance with the relationship of running economy [68]. Likewise, preferred race distance (whether HM, M/UM, or 10 km) is a key factor that has been shown to significantly influence training behavior [69], which therefore significantly affects best race time performance and vice versa [41]. The present dietary subgroups, however, are comparably distributed by preferred race distance. In addition, across dietary subgroups, the subjects have completed a similar number of races, which shows that they have similar psychological backgrounds of experience racing in endurance running events [70]. Furthermore, the runners of each dietary subgroup have been comparably successful in finishing their planned races over the last two years for HM, M, and UM events, which indicates an analogous level of recent racing activity and overall dedication to running [71]. While the present study did not include race-specific variables related to the best time performance results, such as the environmental conditions [3], the subjects did report comparable preferences for racing season across dietary subgroups. In addition, the subjects were predominantly from Germany, Austria, and Switzerland—countries with corresponding running cultures and similar environmental possibilities for running and racing [36]. One significant difference in running history was identified across the dietary subgroups—complete years of active running—showing that the subjects following omnivorous diets completed significantly more years. As the omnivorous diet is the most prevalent in the world, this result is likely due to the primary sample (recreational distance runners) and secondarily to dietary behavior change and the adoption of a vegetarian or vegan diet in adulthood [31,35]. However, this finding is specifically related to the completed years of running without taking a break for any reason (such as focused performance in another sport) and is not equivalent to the total years of running or having a greater training background. 

Regarding the runners’ best performances considering race time, no significant differences were found between subjects following omnivorous, vegetarian, or vegan diets for any long distance (HM, M, or UM races). This finding is rather consistent with previous research showing that there is no running performance difference based on diet type with a general sample of recreational athletes [33]. However, significant differences in running performance by diet type have been identified previously [18,34,35,36,37], which makes the interpretation of the results more complex. Likewise, there is anecdotal evidence of distance runners who adopted a vegan diet winning competitions alongside the claim that their dietary change gives them a substantial edge in performance [16,24,37]. These outcomes may be related to vegan athletes with high nutritional competence (especially tailored dietary strategies at race-day, pre-, in-, and post-race) [27,34,72]. Regarding the UM distance, it was possible for the participants to report their best time in completing any distance of 50 km or greater. Therefore, the precise UM race distance was not considered in this investigation. However, the mean time differences appear rather drastic, although not statistically significant, suggesting that the vegan UM runners (454 min ± 270) were racing at shorter UM distances [73] compared to the omnivores (705 min ± 498) and vegetarians (829 min ± 806).

Importantly, the present investigation included several confounders within multivariate linear regression analyses to bridge the gap between the scientific evidence on the controversial topic at hand [32,33]. The subjects’ running history (explaining 16% of the variance), training behavior (explaining 22% of the variance), and racing experience (explaining 14% of the variance) were each insignificant in mediating performance differences in the best time of HM and M races. Therefore, the present investigation verified the hypothesis that there is no difference in best half-marathon and marathon time performance between recreational long-distance athletes based on adhering to general omnivorous, vegetarian, or vegan diets, which may be attributed to the cross-sectional study design [32]. It appears there are many complex and interwoven factors of best time race performance other than training behaviors (e.g., genetics, performance-enhancing drugs, or even race day strategies) that are challenging to assess with the survey method [33,50], and especially for performance-motivated runners with the goal of a specific finishing time or ranking, thus pointing in the direction of beta error [27,34,39,54]. 

The subjects following an omnivorous diet had the highest BMI (22.6 kg/m^2^), which likely contributes to slower racing times due to a higher energy cost [56]. On the other hand, there were far more females than males following vegan diets in the present sample, and it is well-known that males have a natural running performance advantage even with a significantly higher BMI [48] likely due to differences in body weight, overall body size, anatomical proportions (leg and foot size), muscle mass and fat distributions, and/or iron levels [58,74,75,76,77]. In addition, the subjects following omnivorous diets were the most competition-focused regarding their current running motivation, which is in line with the sex difference identified and previous research showing that males are more competitive [78]. Considering that no performance difference was observable in favor of the omnivores due to the sex difference [48,58,74,75,76,77], the present results are particularly interesting, pointing to a running performance advantage for females following a vegan diet, which has been suggested by previous research [18]. This finding may be due to several health-related factors when following a vegan diet, including but not limited to lower lean body mass accompanied by low fat mass [18,56,79], lesser inflammation [80], greater complex carbohydrate availability [81,82], the lack of heme iron consumption [66,83], lower blood viscosity [84], and/or enhanced vascular function [17,34,85]. 

The properties of the vegan diet may help to explain why no performance difference between diet types has been identified in the males of previous research [32,33,36,37,86] or this investigation. According to the vegan diet type definition used for this study [21], classification required only the lack of consumption of any animal-based food product for four weeks before participation. The general vegan diet’s relationship to health may therefore be unlimited [86]. Whereas a low-fat whole food plant-based diet—a specific vegan diet type—appears to be more limited regarding the direction of the effect on health outcomes [21,86,87,88,89,90,91]. In addition, as physical activity (e.g., running) and sports (e.g., HM or M racing) are considered medicine for sedentary populations [5,12,13], this health factor likely contributes to a marked increase in individual health in the long term. Thus, the mitigating effect of the recreational endurance runner lifestyle (e.g., health conscious, highly physical active) also likely contributes to a lack of significant differences found in best time race performance based on diet type [44,45,46,47]. 

As we identified a considerable variation of 14–22% in our best time performance models, future research on distance running performance in recreational athletes must consider the impact of personal training behavior when comparing diet type with a minimum of eight areas from medium- to high-intensity interval training, pacing, specific competition training, running distance at a low-intensity, support by a qualified sport professional, total periodization duration, and the experience of completing HM and M races. In addition to other performance-influencing factors, the following variables should also be considered in future research: duration of diet type adherence, health status (including nutritional status, disease prevalence), proportion of total daily calories (especially from macronutrients), and the proportion of whole plant foods to processed plant foods. 

The present investigation has limitations. First, the self-reporting feature and cross-sectional study design allow for a wide variation of results, and no causation can be determined. However, controls were implemented as questions in different areas of the survey to reduce the likelihood of misreporting and the best race times were retrospectively verified. In addition, many factors could not be considered in this investigation that are known to impact running performance, and most of the subjects were Western Europeans. Therefore, caution is advised when interpreting the best time performance results between diet types. Nevertheless, the NURMI study is Europe’s largest running study ever conducted, which included a comprehensive outline of runner training behavior and racing experience variables never considered together in previous dietary subgroup comparisons. Another limitation is the unbalanced sex and BMI distribution across dietary subgroups, which may have partially influenced the findings. However, the dietary samples appear to be mostly homogenous regarding their training and racing backgrounds. Lastly, the minimum duration required for the respective classification of vegetarian or vegan dietary adherence was rather low (4 weeks) in order to ensure a large sample, but it is recommended that a 16-week minimum be introduced for enrolling vegetarian or vegan dietary subgroups. While the NURMI study has limitations, it is clear from the results that diet type cannot be ignored in the scientific discussion of top running performance.

## 5. Conclusions

General diet type categories (omnivorous, vegetarian, vegan) appear to make no difference in the best time running performance of HM and M races among recreational athletes, even when considering various training behaviors and experience confounders. The minimal impact of diet type on running performance for highly nutritionally competent (especially tailored dietary strategies at race-day, pre-, in-, and post-race times) athletes may, however, make the difference in finishing a long-distance running event and especially in achieving a higher place (e.g., first place), or even not. The results show that more experimental research is needed to better understand the potential relationship of vegan vs. non-vegan diets with endurance performance.

## Figures and Tables

**Table 2:** Multiple Linear Regression Analyses on Running History, Training Behaviors, and Racing Experiences.

	b	95% CI	*p*-Value	Adjusted R^2^
**Running History**				
Intercept	65.7	79.1, 52.4	<0.001	
Vegetarian diet	−1.66	8.27, −11.6	>0.05	
Vegan diet	0.491	8.74, −7.76	>0.05	0.16
Complete years running	0.928	1.43, 0.43	<0.001	
Age at first race (years)	−0.882	−0.55, −1.22	<0.001	
**Training Behaviors**				
Intercept	27.5	39.9, 15.0	<0.001	
Vegetarian diet	−0.495	9.51, −10.5	>0.05	
Vegan diet	1.85	10.0, −6.33	>0.05	
Training type C	6.04	10.1, 1.94	<0.01	0.22
Training type D	−2.12	1.72, −5.97	>0.05	
Training type A: weekly km	0.293	0.47, 0.12	<0.001	
Professional support	10.2	20.7, −0.35	>0.05	
Training duration	−1	0.96, −2.97	>0.05	
**Racing Experiences**				
Intercept	38.6	49.3, 27.9	<0.001	
Vegetarian diet	−0.716	9.22, −10.7	>0.05	
Vegan diet	1.4	9.59, −6.78	>0.05	
Completed HM and M races	0.532	1.04, 0.02	<0.05	0.14
Proportion HM/M to other races	−0.077	0.08, −0.23	>0.05	
Number of HM completed	0.53	2.43, −1.37	>0.05	
Number of M completed	1.9	3.9, −0.11	>0.05	

Note. b = estimate (marginal effects); CI = confidence interval; km—kilometers; HM—half-marathon; M—marathon.

## Data Availability

The data sets generated during and/or analyzed during in the current study are not publicly available but may be made available upon through reasonable request. If desired, subjects will receive a brief summary of the results of the NURMI Study.
